# The Role of Endoplasmic Reticulum Stress and Unfolded Protein Response in Gynecological Cancers: A Narrative Review

**DOI:** 10.7759/cureus.94434

**Published:** 2025-10-13

**Authors:** Stefanos Flindris, Konstantinos Flindris, Spyros Foutadakis, Michail Kalinderis, Alexandros Traianos, Vassiliki I Kigka, Freideriki Nteka, George Mpourazanis, Ioanna Styliara, Effrosyni Styliara, Panagiotis Tsirkas, Stamatios Petousis, Chrysoula Margioula-Siarkou, Iordanis Navrozoglou

**Affiliations:** 1 2nd Department of Obstetrics and Gynecology, Aristotle University School of Medicine, Ippokratio General Hospital, Thessaloniki, GRC; 2 Department of Ophthalmology, University General Hospital of Ioannina, Ioannina, GRC; 3 Center of Basic Research, Biomedical Research Foundation Academy of Athens, Athens, GRC; 4 Department of Obstetrics and Gynecology, School of Medicine, University of Ioannina, Ioannina, GRC; 5 Department of Cardiac Surgery, Univeristy General Hospital of Ioannina, Ioannina, GRC; 6 Department of Obstetrics and Gynecology, General Hospital of Ioannina G. Hatzikosta, Ioannina, GRC; 7 Department of Obstetrics and Gynecology, School of Medicine, University of Patras, Patras, GRC; 8 Department of Radiology, University General Hospital of Ioannina, Ioannina, GRC; 9 Department of Gynecology and Obstetrics, Thessaloniki University, Thessaloniki, GRC

**Keywords:** atf6/grp78, cervical cancer, endometrial cancer, er stress, gynecologic oncology, ire1/xbp1, ovarian cancer, perk/eif2α/atf4, targeted therapy, unfolded protein response

## Abstract

The unfolded protein response (UPR) mediated by IRE1/XBP1, PERK/eIF2α/ATF4, and ATF6/GRP78 governs tumor adaptation to hypoxia, nutrient deprivation, and therapy stress. In gynecologic malignancies (ovarian, endometrial, cervical), persistent endoplasmic reticulum (ER)-stress signaling underlies proliferation, invasion, immune evasion, and treatment resistance but also creates druggable liabilities. We conducted a narrative review of peer-reviewed literature indexed in MEDLINE (PubMed), Embase, Scopus, and Web of Science from inception through 13 September 2025. Search terms combined ER-stress/UPR pathways with gynecologic cancers. Eligible records included recent and high-quality preclinical, translational, and clinical studies, clinical trials, and high-quality reviews focused on UPR biology, biomarkers, or therapeutics. Data were synthesized by disease site and UPR branch. Across tumor types, UPR activation correlates with aggressive phenotypes and poorer outcomes. In epithelial ovarian cancer, GRP78/ATF6/PERK overexpression associates with inferior survival and chemoresistance, supporting their utility as biomarkers and therapeutic targets. Endometrial cancer demonstrates UPR gene-signature stratification of prognosis and immune infiltration, suggesting risk-adapted strategies. Cervical cancer leverages PERK/IRE1 signaling for therapy tolerance and dormancy. The tumor-immune interface is UPR-sensitive: CHOP and myeloid IRE1α signaling can dampen antitumor immunity, providing a rationale to pair UPR modulation with immunotherapy. Therapeutically, IRE1 RNase inhibitors (e.g., MKC-8866, B-I09), PERK/EIF2AK3 pathway modulators, protein-disulfide isomerase inhibition, and agents that trigger irrecoverable ER stress show preclinical efficacy, including synergy with platinum, poly (ADP-ribose) polymerase (PARP) inhibitors, HDAC6 blockers, and PD-1 inhibitors. Early clinical efforts investigating ER-stress-modulating combinations in platinum-resistant ovarian cancer highlight translational promise but remain preliminary. Thus, ER-stress/UPR signaling is a convergent, targetable axis in gynecologic cancers. Priorities include validating UPR-based prognostic signatures, defining context-specific vulnerabilities (e.g., genotype-informed IRE1/XBP1 dependence), and executing biomarker-driven clinical trials that combine UPR-targeted agents with standard chemotherapy, PARP inhibition, and immunotherapy to overcome resistance and improve patient outcomes.

## Introduction and background

The unfolded protein response (UPR) is an adaptive pathway activated when the endoplasmic reticulum (ER) experiences stress due to misfolded or excess proteins. This response is mediated by three key ER transmembrane sensors: protein kinase RNA-like ER kinase (PERK or EIF2AK3), inositol-requiring enzyme-1 (IRE1), and activating transcription factor-6 (ATF6) [1]. Under normal conditions, the chaperone GRP78 (78 kDa glucose-regulated protein or BiP: binding immunoglobulin protein) binds to these sensors, keeping them inactive [2]. However, when unfolded proteins accumulate due to stressors such as hypoxia, nutrient deprivation, or oncogene-driven protein synthesis, GRP78 dissociates, leading to sensor activation [2].

Initially, the UPR aims to restore proteostasis by reducing protein translation, upregulating chaperones, and enhancing protein degradation. However, when ER stress is prolonged or severe, the UPR may switch to pro-apoptotic programs; for example, this shift between adaptive and terminal UPR is crucial in determining cell fate [3]. In cancer, solid tumors often experience significant ER stress due to their hostile microenvironment, including hypoxia, glucose deprivation, oxidative stress, and low pH, disrupting protein folding [4]. Frequently, cancer cells exhibit constitutive activation of UPR pathways to manage chronic stress. One notable feature is that the overexpression of the UPR initially serves a protective homeostatic function; it is hijacked in cancer to promote tumor growth, survival, and resistance to treatment [5]. Consequently, cancer cells exploit the protective aspects of the UPR to survive and proliferate.

Upon activation, IRE1 oligomerizes and autophosphorylates, initiating its endoribonuclease activity, which leads to the unconventional splicing of XBP1 mRNA, producing XBP1s, a potent transcription factor. XBP1s enhances the ER’s protein-folding capacity and supports ER-associated degradation, aiding in stress resolution. In cancer, the IRE1-XBP1 pathway is essential for tumor cell survival under stress and may promote tumor aggressiveness [6,7]. For instance, silencing XBP1 impairs hypoxic tumor cell survival and inhibits growth in xenograft models. Moreover, XBP1 is hyperactivated in triple-negative breast cancer (TNBC), promoting tumor progression and correlating with poorer relapse-free survival [8].

PERK (protein kinase RNA-like ER kinase), another critical UPR component, influences tumor biology by modulating protein synthesis and transcriptional programs. Upon activation, PERK also upregulates stress-adaptation proteins, such as ATF4 and Nrf2, promoting cell survival. Studies have shown that PERK activation helps tumor cells survive under hypoxic conditions, enhancing their migration and metastatic potential [9]. For example, PERK activation induces the metastasis-associated gene LAMP3, linking UPR activation to increased metastatic potential. Additionally, PERK activity enables cancer cells to resist oxidative DNA damage, further enhancing tumor growth [10]. Both the IRE1 and PERK branches of the UPR are essential for cancer cells to adapt to stress, supporting survival, proliferation, and malignancy. Their frequent activation in gynecological cancers underscores their potential as therapeutic targets in cancer treatment [8,10].

In aggregate, UPR signaling provides a coherent lens on how solid tumors survive chronic stress. In gynecologic cancers, distinctive pressures, such as hormone-regulated secretory programs, intermittent hypoxia in the tumor microenvironment, metabolic reprogramming, and the disruption of proteostasis by HPV in cervical cancer, impose persistent ER load. These conditions heighten dependence on the PERK, IRE1, and ATF6 pathways, shaping proliferation, immune evasion, and tolerance to therapy. This review centers on ovarian, endometrial, and cervical cancers and outlines key biomarkers with prognostic relevance, mechanistic connections to the tumor-immune interface, and therapeutic strategies with associated trial-design considerations that aim to translate this biology into patient benefit.

## Review

Methods

This narrative review synthesizes peer-reviewed literature on ER stress and the UPR (IRE1/XBP1, PERK/eIF2α/ATF4, ATF6/GRP78) in gynecologic cancers (ovarian, endometrial, cervical). We searched MEDLINE (PubMed), Embase, Scopus, and Web of Science from inception to 13 September 2025, screened reference lists of key papers, and restricted inclusion to English-language publications. Eligible studies comprised original preclinical, translational, and clinical research, clinical trials, and high-quality reviews addressing ER-stress/UPR biology, biomarkers, or therapeutics in these cancers; we excluded non-peer-reviewed sources, conference abstracts without full text, and single case reports lacking mechanistic insight. We applied a structured, design-specific appraisal to document study design, key risks of bias, and evidentiary weight across in vivo, cell-based, observational clinical studies, and clinical trials. Each study was assigned an evidence tier (mechanistic, preclinical efficacy, translational biomarker, or clinical efficacy); higher-tier evidence guided the narrative synthesis, and study limitations were considered in interpretation. Two reviewers independently screened titles/abstracts and full texts, resolving discrepancies by consensus. We extracted data on cancer type or model, UPR arm(s), interventions, and main outcomes, and synthesized findings by pathway branch and disease site. No meta-analysis was planned.

The role of ER stress and the UPR in ovarian cancer

ER stress and the UPR play critical roles in ovarian cancer progression, chemoresistance, and immune evasion. In high-grade serous ovarian carcinoma (HGSOC), key UPR regulators, such as IRE1 and PERK, enable tumor adaptation to hypoxia and metabolic stress [11]. Chronic ER stress promotes autophagy and apoptosis resistance, contributing to platinum-based chemotherapy failure. Furthermore, UPR activation modulates immune infiltration within the tumor microenvironment, influencing responsiveness to immunotherapy [11]. Consequently, targeting ER stress pathways, either through direct inhibition of UPR sensors or in combination with conventional therapies, represents a promising strategy to improve ovarian cancer outcomes.

Overexpression of GRP78, ATF6, and PERK in epithelial ovarian cancer (EOC), with elevated levels correlating with poorer survival (GRP78: p<0.0001; GRP78/PDI: p=0.03) [12]. Notably, high GRP78 and PDI expression in HGSOC predicts worse prognosis, suggesting their utility as dual prognostic biomarkers [11,13]. Although the IRE1-XBP1 axis has been implicated in epithelial-mesenchymal transition (EMT) and invasion in colorectal cancer, paradoxically, low XBP1 mRNA levels in serous ovarian cancer also predict poor outcomes, underscoring the context-dependent roles of distinct UPR branches [12,13].

Recent evidence reveals a bidirectional regulatory loop between PERK and IRE1 under chronic ER stress. In triple-negative breast cancer cells, PERK inhibition with Amgen 44 reduces IRE1 expression, whereas active IRE1 sustains PERK protein levels via XBP1s-mediated transcriptional upregulation of PERK. XBP1s binding to the PERK promoter thus establishes a feed-forward mechanism essential for cell survival under prolonged stress [14]. This interdependence highlights the rationale for dual-targeting strategies in UPR-addicted tumors, including ovarian and endometrial cancers [14].

Somatic mutations in UPR-related genes are prevalent across gynecological malignancies. In ovarian cancer, IRE1α mutations often abrogate RIDD activity while retaining XBP1 splicing, conferring survival advantages [15]. Similarly, XBP1 mutations in multiple myeloma and PERK/ATF6 alterations in lung and renal cancers suggest tissue-specific mutational landscapes that may influence therapeutic resistance [16,17].

Mechanistically, IRE1 is a type I transmembrane kinase/endonuclease that is activated by ER stress to splice XBP1 mRNA, promoting adaptive survival pathways [18]. Prolonged IRE1 activation induces regulated IRE1-dependent decay (RIDD) of secretory mRNAs and engages the TRAF2/ASK1/JNK axis, downregulating BCL-2 family proteins and activating caspase‑12 to trigger apoptosis [19]. Beyond tumor cells, the IRE1α-XBP1 axis also governs cancer stemness and reprograms the lipid metabolism of tumor-infiltrating dendritic and T cells, rendering it a compelling immunotherapeutic target [6,20-22].

PERK activation attenuates global protein synthesis through phosphorylation of eIF2α, alleviating ER burden. While transient PERK signaling fosters survival, sustained activation induces ATF4 [23]. PERK also phosphorylates NRF2, enhancing antioxidant responses and ERO1α-mediated protein folding, which supports tumor growth [23]. Nevertheless, CHOP-driven apoptosis remains pivotal in ER stress-mediated cell death in ovarian cancer [21].

Moreover, ovarian cancer cells activate UPR pathways to resist chemotherapy and hypoxia. Tunicamycin-induced ER stress sensitizes cells to platinum agents by activating apoptotic cascades. Moreover, the PERK-eIF2α-ATF4-CHOP axis promotes autophagy-mediated chemoresistance [24]. Under hypoxic conditions, PERK deficiency impairs redox homeostasis and LC3 turnover, whereas IRE1-driven XBP1 splicing enhances hypoxia tolerance [25].

Mutations in ARID1A are present in over 50% of ovarian clear cell carcinomas (OCCC) and repress the IRE1α-XBP1 axis, sensitizing tumors to IRE1α inhibitors in combination with HDAC6 inhibition [26]. In the tumor microenvironment, activation of IRE1α in dendritic cells induces lipid metabolic dysfunction and impairs T‑cell priming, whereas XBP1 deletion in T cells enhances interferon-γ production and prolongs survival in ovarian cancer models [27,28].

Furthermore, constitutive XBP1 activation in tumor-associated dendritic cells disrupts lipid metabolism and antigen presentation, blunting T-cell priming and accelerating ovarian cancer progression. Complementarily, malignant ascites imposes ER stress in tumor-infiltrating T cells and triggers IRE1α-XBP1 activation that limits mitochondrial respiration and IFN-γ; genetic or pharmacologic blockade restored effector function in patient T cells and improved survival in mouse models. These data provide a mechanistic rationale for pairing IRE1/XBP1 inhibitors with immune checkpoint blockade in gynecologic tumors [29].

High CHOP expression in intratumoral CD8+ T cells correlates with poor clinical outcomes by repressing T-bet, thereby limiting cytotoxic function. Inhibition of CHOP or upstream PERK signaling may restore T-cell effector activity and improve patient survival [30]. Similarly, neutrophil-specific deletion of IRE1α delays tumor progression, augments T‑cell-mediated control, and sensitizes tumors to PD-1 blockade [11,31].

Therapeutically, combination regimens that provoke ER stress, such as the WEE1 inhibitor AZD1775, activate both PERK and IRE1α in p53-mutant ovarian cancer cells [32]. Likewise, inhibition of stearoyl-CoA desaturase 1 (SCD1) induces ER stress-mediated apoptosis selectively in cancer cells, an effect reversed by exogenous oleic acid [33]. These findings underscore the dual roles of UPR sensors in mediating survival and death and support the development of dual-targeted therapies to exploit ER stress vulnerabilities in ovarian cancer.

The role of ER stress and the UPR in endometrial cancer

Endometrial cancer is the most prevalent malignancy of the female genital tract. Established risk factors include obesity, diabetes, unopposed estrogen therapy, polycystic ovarian syndrome, and a Westernized lifestyle [34]. Emerging evidence implicates ER stress and its attendant UPR in the progression, metastasis, and therapy resistance of endometrial tumors [35].

The UPR comprises three primary signaling branches initiated by the ER-resident sensors IRE1, PERK, and ATF6. In endometrial cancer, dysregulated activation of these pathways fosters key malignant phenotypes. IRE1-mediated splicing of XBP1 mRNA enhances angiogenesis and immune evasion, while PERK-driven phosphorylation of eIF2α and subsequent ATF4 activation bolster antioxidant defenses and protein homeostasis, thereby promoting cell survival under stress [36]. Notably, pharmacological blockade of IRE1-XBP1 or PERK-eIF2α/ATF4 axes has demonstrated anti-tumor efficacy in preclinical models, underscoring their therapeutic potential [28,37]. Immunohistochemical analyses by Bifulco et al. revealed upregulated expression of key ER stress markers GRP78, ATF6, and CHOP in endometrial adenocarcinoma [38].

In addition, pro-apoptotic UPR signaling is chiefly mediated via CHOP and p53-dependent mechanisms. CHOP expression is significantly elevated in invasive endometrial lesions relative to normal endometrium, correlating with markers of poor prognosis [39]. ER stress also modulates p53 target genes, such as CDKN1A and MDM2, thereby influencing the DNA damage response and genomic stability in endometrial cancer cells [36,40]. Although direct studies in endometrial cancer remain limited, analogies with breast cancer models highlight potential UPR-mediated mechanisms. PERK and ATF6 have been implicated in tumor grade and stage via regulation of stemness factors, such as SOX2 [41]. Moreover, hypoxia-induced IRE1-XBP1 signaling, known to drive angiogenesis and survival in breast tumors, is likely operative in endometrial carcinogenesis. PERK-dependent miR-211 production further suppresses CHOP expression, enabling tumor cells to withstand nutrient deprivation [42].

Risk models incorporating ER stress-related genes have shown prognostic relevance in endometrial cancer. A recent study identified a four-gene signature (TRIB3, CREB3L3, XBP1, PPP1R15A) that stratifies patients by survival outcomes and correlates low-risk status with increased infiltration of CD8⁺ T cells, dendritic cells, and elevated PD-L1 and CTLA-4 expression [43]. Similarly, a model featuring MYBL2, RADX, RUSC2, and CYP46A1 reveals that low-risk patients exhibit greater sensitivity to dactinomycin and trametinib [43,44].

Of note, targeting UPR components has emerged as a promising strategy in preclinical studies. The proteasome inhibitor carfilzomib induces ER stress-mediated apoptosis in endometrial carcinoma cells, marked by dose-dependent increases in GRP78. PERK activation may confer a survival advantage under transient stress [35].

Comprehensive elucidation of context-specific UPR dynamics in endometrial cancer is essential for the rational design of UPR-targeted therapies. ER stress and the UPR are integral to endometrial cancer pathobiology, influencing tumor progression, immune interactions, and treatment responsiveness. Prognostic models incorporating UPR-related genes hold promise for patient stratification, while therapeutic interventions targeting ER stress pathways warrant further clinical investigation.

The role of ER stress and the UPR in cervical cancer progression

ER stress is a critical driver of cervical cancer development, treatment resistance, and immune modulation. Persistent activation of the UPR, particularly via the PERK and IRE1 pathways, correlates with poor prognosis and enhanced tumor survival under hypoxic conditions. Moreover, ER stress-induced dormancy enables cervical cancer cells to evade radiotherapy and chemotherapy, while UPR-mediated immune suppression attenuates antitumor responses. Disrupting these survival mechanisms, such as inhibiting PERK-mediated translational attenuation or blocking IRE1-driven cytokine production, holds promise for sensitizing tumors to existing therapies and improving patient outcomes [37,45,46].

ER stress also plays a pivotal role in chemoresistance. In HeLa and CaSki cell lines, both paclitaxel and cisplatin trigger autophagy and ER stress-mediated apoptosis [47]. Paclitaxel-induced cell death involves Beclin-1 and LC3; when autophagy is inhibited, GRP78 is upregulated by paclitaxel exposure [47]. Similarly, cisplatin upregulates GRP78 [47,48]. Dysregulation of UPR sensors further contributes to cervical cancer progression. Fenretinide induces apoptosis via the PERK-eIF2α-caspase-12 axis, underscoring ER stress as a targetable vulnerability. In contrast, ATF6 promotes epithelial-mesenchymal transition (EMT) by downregulating E-cadherin and upregulating Snail and vimentin through MAPK signaling, thereby enhancing invasiveness [46].

Importantly, ER stress-related proteins also drive tumor migration and therapy resistance. SEC62, a mediator of ER-phagy, is amplified in high-grade cervical lesions and correlates with increased aggressiveness; silencing SEC62 in HeLa cells markedly inhibits migration [49]. Radiotherapy-induced activation of ERN1 (IRE1) has been implicated in cancer cell dormancy, enabling treatment evasion and recurrence in cervical and ovarian cancers [37]. Finally, Yip1A, a multipass ER membrane protein, has emerged as a key regulator of ER stress in cervical cancer. Under chronic ER stress, Yip1A enhances IRE1 phosphorylation and PERK transcriptional activity, upregulating anti-apoptotic proteins and autophagy factors to promote cytoprotective autophagy and apoptosis resistance in HeLa and CaSki cells. Depletion of Yip1A, however, induces apoptosis, identifying it as a critical prosurvival modulator and potential therapeutic target [24,50].

Prognostic signatures and radiotherapy resistance

ER stress-related genes have emerged as important components of both prognostic modeling and radiotherapy resistance in cervical cancer. In a comprehensive analysis of 255 ER stress-associated genes, Liu et al. identified 25 whose expression correlated significantly with patient survival [51]. Their multivariate risk model singled out FOXRED2 expression and lymph node metastasis (N stage) as independent predictors of poor outcome. Patients classified as low-risk not only exhibited better overall survival but also showed higher levels of tumor-infiltrating immune cells, particularly CD8⁺ T cells and regulatory T cells [45]. Moreover, radiotherapy itself modulates the expression of ER stress genes, with IRE1 playing a central role in mediating radiation-induced mucosal toxicity. Given that ER stress-induced dormancy allows cervical cancer cells to withstand radiotherapy and chemotherapy, these findings suggest that targeting key UPR mediators could sensitize tumors to standard treatments [26,52].

Therapeutic advances

Several clinical trials are now exploring ER-stress modulation as a therapeutic strategy in cancer. For example, NCT04648033 is evaluating the ER-stress inducers auranofin and disulfiram in platinum-resistant ovarian cancer, while NCT03456700 combines the procaspase-activating compound PAC-1 with gemcitabine to potentiate ER-stress-mediated apoptosis [53]. Emerging small-molecule inhibitors include the IRE1 RNase blockers B-I09 and MKC-8866, as well as PERK inhibitors such as BOLD-100, which induces ER stress via reactive oxygen species and calcium release, and is under investigation in platinum-resistant ovarian cancer [26,54].

The IRE1 and future biomarker-driven strategies will be essential to identify patients most likely to benefit from UPR-targeted therapies in gynecological malignancies. In cervical cancer, Adamczyk-Grochala et al. [55] demonstrated that doxorubicin (DOX) treatment activates ER-stress pathways, particularly loss of DNMT2/TRDMT1, also impaired GRP78/BiP chaperone function, leading to misfolded protein accumulation, caspase-3 activation, and DNA damage [55]. Although DOX triggers all three UPR sensors (PERK, IRE1, and ATF6), PERK activation predominates; thus, targeting DNMT2/TRDMT1 may sensitize cervical cancer cells to DOX by disabling this survival mechanism [55,56].

Furthermore, Bonsignore et al. further highlighted the role of PERK and IRE1 pathways in therapy resistance across tumor types. In lung cancer, high ATF4 (downstream of PERK) levels correlate with cisplatin resistance, whereas ATF4 knockout enhances drug sensitivity [57]. Radiotherapy activates the PERK-eIF2α-ATF4-LAMP3 axis in breast cancer, and PERK inhibition increases radiosensitivity. Similarly, overexpression of XBP1 (an IRE1 effector) promotes hormone-independent growth and antiestrogen resistance in breast cancer [58]. ER-stress inducers such as tunicamycin can either sensitize tumors to chemotherapy (e.g., cisplatin in ovarian cancer) or induce resistance via GRP78 [12,24]. In ovarian and related gynecological malignancies, several small-molecule inhibitors targeting the IRE1α-XBP1 arm of the UPR have been developed to undermine pro-survival signaling and overcome chemoresistance. The RNase-specific inhibitor MKC8866 prevents [32]. Similarly, B-I09 blocks IRE1α’s endonuclease activity, suppresses protumorigenic XBP1s signaling, and exerts additive antitumor effects when combined with PD-1 blockade or HDAC6 inhibition in ARID1A-mutant and CARM1-overexpressing ovarian clear-cell carcinoma models [27].

In addition, convergent data nominate the IRE1α-XBP1 arm as a tractable dependency in ovarian cancer. In ARID1A-mutant ovarian clear cell carcinoma, loss of ARID1A de-represses IRE1α-XBP1 signaling and creates vulnerability to IRE1α RNase blockade: B-I09 curtailed growth in orthotopic xenografts, patient-derived xenografts, and conditional Arid1a/Pik3ca models; Xbp1 knockout prolonged survival; and B-I09 synergized with HDAC6 inhibition in vivo [26]. In CARM1-expressing high-grade serous ovarian cancer, CARM1 physically cooperates with XBP1s to determine pathway reliance; accordingly, IRE1α/XBP1s inhibition was effective in vitro and in orthotopic/PDX systems, potentiated anti-PD-1 activity in an immunocompetent model, and showed acceptable tolerability [59]. Extending beyond tumor-intrinsic biology, ovarian cancer ascites activate IRE1α-XBP1 in T cells, diminishing mitochondrial fitness and IFN-γ; genetic or pharmacologic blockade restored human T-cell function ex vivo, and T-cell-specific Xbp1 deletion enhanced antitumor immunity and survival in mice [60]. Consistent with a therapy-resistance role, an IRE1α inhibitor reduced cisplatin resistance and re-sensitized ovarian cancer models in vitro and in vivo [15].

Beyond IRE1α, additional UPR nodes shape treatment response. In high-grade serous disease, a p38-ATF6-AP-1 program emerges in PARP inhibitor-resistant models; p38 inhibition lowered nuclear ATF6, depleted RAD51 foci, increased DNA damage, and reduced tumor burden in a PDX, supporting ATF6-directed combinations to overcome PARPi resistance [61]. In endometrial cancer, GRP78/BiP functionally links to cisplatin resistance: cisplatin induces GRP78, GRP78 knockdown augments cisplatin-mediated death, and AKT inhibition down-modulates GRP78 and mitigates chemoresistance; estrogen-induced GRP78 further modulates sensitivity [62]. Finally, disruption of ER proteostasis via protein-disulfide isomerase blockade shows ovarian-specific signals: the covalent PDI inhibitor PACMA-31 impaired survival of human ovarian cancer cells, suppressed in vivo growth without overt toxicity, and re-sensitized A2780cis models to cisplatin [63].

Together, these pharmacological advances illustrate a dual strategy: disabling IRE1α-mediated adaptive UPR signaling while exploiting PERK-driven apoptotic pathways. By carefully optimizing the dosing and scheduling of IRE1 versus PERK modulators, either as monotherapies or in combination with chemotherapy and immunotherapy, these agents hold promise for more durable responses in ovarian, cervical, and endometrial cancers.

Strengths and limitations

The strengths of the current review lie in bridging basic and translational evidence, highlighting emerging biomarkers and therapeutic opportunities that could inform future research and patient care. Nonetheless, as a narrative review, it is subject to potential selection bias and lacks the quantitative rigor of a systematic analysis, while variability across experimental models and reporting standards may limit the generalizability of some conclusions.

## Conclusions

In conclusion, current evidence indicates that ER stress signaling through the PERK with PERK-ATF4, ATF6, and IRE1-XBP1 reprograms proteostasis, metabolism, and cell fate in ovarian, endometrial, and cervical cancer, with "context-dependent" effects on proliferation, survival, angiogenesis, invasion, and immune evasion. Sustained pathway activation associates with adverse clinicopathologic features and resistance to platinum agents, taxanes, and PARP inhibition in preclinical systems. Genetic or pharmacologic interruption of IRE1 RNase activity, ATF6 processing, or eIF2α phosphorylation restores drug sensitivity, restricts tumor growth in orthotopic and patient-derived xenograft models, and improves antitumor T cell function. These data justify biomarker-guided evaluation of unfolded protein response modulators, including selective IRE1 RNase inhibitors, PERK pathway regulators, and stress sensitizers, alone or combined with DNA damage response inhibitors and immune checkpoint therapy. Immediate translational priorities include standardized assays of pathway activity in clinical specimens and prespecified predictive cutoffs. Early-phase trials should incorporate pharmacodynamic verification of on-target effects in tumor and immune compartments while monitoring toxicity in secretory organs. A rigorous program along these lines can determine whether targeting stress adaptation achieves meaningful gains in survival and quality of life for patients with recurrent or refractory gynecologic malignancies.

## References

[1] Nie Z, Chen M, Wen X (2021). Endoplasmic reticulum stress and tumor microenvironment in bladder cancer: the missing link. Front Cell Dev Biol.

[2] Chipurupalli S, Kannan E, Tergaonkar V, D'Andrea R, Robinson N (2019). Hypoxia induced ER stress response as an adaptive mechanism in cancer. Int J Mol Sci.

[3] Cherubini A, Zito E (2022). ER stress as a trigger of UPR and ER-phagy in cancer growth and spread. Front Oncol.

[4] Fernandez PM, Tabbara SO, Jacobs LK (2000). Overexpression of the glucose-regulated stress gene GRP78 in malignant but not benign human breast lesions. Breast Cancer Res Treat.

[5] Hsu SK, Chiu CC, Dahms HU (2019). Unfolded protein response (UPR) in survival, dormancy, immunosuppression, metastasis, and treatments of cancer cells. Int J Mol Sci.

[6] McGrath EP, Logue SE, Mnich K, Deegan S, Jäger R, Gorman AM, Samali A (2018). The unfolded protein response in breast cancer. Cancers (Basel).

[7] Ogura J, Yamanoi K, Ishida K (2024). A stearate-rich diet and oleate restriction directly inhibit tumor growth via the unfolded protein response. Exp Mol Med.

[8] Chen X, Iliopoulos D, Zhang Q (2014). XBP1 promotes triple-negative breast cancer by controlling the HIF1α pathway. Nature.

[9] Wang P, Han L, Yu M, Cao Z, Li X, Shao Y, Zhu G (2021). The prognostic value of PERK in cancer and its relationship with immune cell infiltration. Front Mol Biosci.

[10] Sisinni L, Pietrafesa M, Lepore S, Maddalena F, Condelli V, Esposito F, Landriscina M (2019). Endoplasmic reticulum stress and unfolded protein response in breast cancer: the balance between apoptosis and autophagy and its role in drug resistance. Int J Mol Sci.

[11] Emmanuelli A, Salvagno C, Hwang SM (2024). High-grade serous ovarian cancer development and anti-PD-1 resistance is driven by IRE1α activity in neutrophils. Oncoimmunology.

[12] Samanta S, Tamura S, Dubeau L (2020). Clinicopathological significance of endoplasmic reticulum stress proteins in ovarian carcinoma. Sci Rep.

[13] Khan AA, Allemailem KS, Almatroudi A, Almatroodi SA, Mahzari A, Alsahli MA, Rahmani AH (2020). Endoplasmic reticulum stress provocation by different nanoparticles: an innovative approach to manage the cancer and other common diseases. Molecules.

[14] Ong G, Ragetli R, Mnich K, Doble BW, Kammouni W, Logue SE (2024). IRE1 signaling increases PERK expression during chronic ER stress. Cell Death Dis.

[15] Lv S, Zhang L, Wu M (2024). IRE1α inhibitor reduces cisplatin resistance in ovarian cancer by modulating IRE1α/XBP1 pathway. Cell Oncol (Dordr).

[16] Storm M, Sheng X, Arnoldussen YJ, Saatcioglu F (2016). Prostate cancer and the unfolded protein response. Oncotarget.

[17] Carrasco DR, Sukhdeo K, Protopopova M (2007). The differentiation and stress response factor XBP-1 drives multiple myeloma pathogenesis. Cancer Cell.

[18] Sakatani T, Maemura K, Hiyama N (2017). High expression of IRE1 in lung adenocarcinoma is associated with a lower rate of recurrence. Jpn J Clin Oncol.

[19] Yuan S, She D, Jiang S, Deng N, Peng J, Ma L (2024). Endoplasmic reticulum stress and therapeutic strategies in metabolic, neurodegenerative diseases and cancer. Mol Med.

[20] Madden E, Logue SE, Healy SJ, Manie S, Samali A (2019). The role of the unfolded protein response in cancer progression: from oncogenesis to chemoresistance. Biol Cell.

[21] Yan T, Ma X, Guo L, Lu R (2023). Targeting endoplasmic reticulum stress signaling in ovarian cancer therapy. Cancer Biol Med.

[22] Zhou P, Wu C, Ma C, Luo T, Yuan J, Zhou P, Wei Z (2023). Identification of an endoplasmic reticulum stress-related gene signature to predict prognosis and potential drugs of uterine corpus endometrial cancer. Math Biosci Eng.

[23] Rozpedek W, Pytel D, Mucha B, Leszczynska H, Diehl JA, Majsterek I (2016). The role of the PERK/eif2α/ATF4/CHOP signaling pathway in tumor progression during endoplasmic reticulum stress. Curr Mol Med.

[24] Fujimoto A, Kawana K, Taguchi A (2016). Inhibition of endoplasmic reticulum (ER) stress sensors sensitizes cancer stem-like cells to ER stress-mediated apoptosis. Oncotarget.

[25] Moon HS, Kim B, Gwak H, Suh DH, Song YS (2016). Autophagy and protein kinase RNA-like endoplasmic reticulum kinase (PERK)/eukaryotic initiation factor 2 alpha kinase (eIF2α) pathway protect ovarian cancer cells from metformin-induced apoptosis. Mol Carcinog.

[26] Zundell JA, Fukumoto T, Lin J (2021). Targeting the IRE1α/XBP1 endoplasmic reticulum stress response pathway in ARID1A-mutant ovarian cancers. Cancer Res.

[27] Salvagno C, Mandula JK, Rodriguez PC, Cubillos-Ruiz JR (2022). Decoding endoplasmic reticulum stress signals in cancer cells and antitumor immunity. Trends Cancer.

[28] Ulianich L, Insabato L (2014). Endoplasmic reticulum stress in endometrial cancer. Front Med (Lausanne).

[29] Cubillos-Ruiz JR, Silberman PC, Rutkowski MR (2015). ER stress sensor XBP1 controls anti-tumor immunity by disrupting dendritic cell homeostasis. Cell.

[30] Cao Y, Trillo-Tinoco J, Sierra RA (2019). ER stress-induced mediator C/EBP homologous protein thwarts effector T cell activity in tumors through T-bet repression. Nat Commun.

[31] Yao J, Ji L, Wang G, Ding J (2025). Effect of neutrophils on tumor immunity and immunotherapy resistance with underlying mechanisms. Cancer Commun (Lond).

[32] Xiao R, You L, Zhang L (2022). Inhibiting the IRE1α axis of the unfolded protein response enhances the antitumor effect of AZD1775 in TP53 mutant ovarian cancer. Adv Sci (Weinh).

[33] Lee J, Jang S, Im J (2024). Stearoyl-CoA desaturase 1 inhibition induces ER stress-mediated apoptosis in ovarian cancer cells. J Ovarian Res.

[34] Makker V, MacKay H, Ray-Coquard I, Levine DA, Westin SN, Aoki D, Oaknin A (2021). Endometrial cancer. Nat Rev Dis Primers.

[35] Zhaozu F, Ke W, Yunfengc M, Wenjuan L (2023). Carfilzomib induces apoptosis of endometrial carcinoma cells by activating endoplasmic reticulum stress. Clin Oncol.

[36] Barua D, Gupta A, Gupta S (2020). Targeting the IRE1-XBP1 axis to overcome endocrine resistance in breast cancer: opportunities and challenges. Cancer Lett.

[37] Ghemrawi R, Kremesh S, Mousa WK, Khair M (2025). The role of ER stress and the unfolded protein response in cancer. Cancer Genomics Proteomics.

[38] Bifulco G, Miele C, Di Jeso B (2012). Endoplasmic reticulum stress is activated in endometrial adenocarcinoma. Gynecol Oncol.

[39] Matsuo K, Gray MJ, Yang DY (2013). The endoplasmic reticulum stress marker, glucose-regulated protein-78 (GRP78) in visceral adipocytes predicts endometrial cancer progression and patient survival. Gynecol Oncol.

[40] Zhou J, Lin Y, Yang X, Shen B, Hao J, Wang J, Wang J (2022). Metabolic disorders sensitise endometrial carcinoma through endoplasmic reticulum stress. Cell Mol Biol Lett.

[41] Yang N, Hui L, Wang Y, Yang H, Jiang X (2014). SOX2 promotes the migration and invasion of laryngeal cancer cells by induction of MMP-2 via the PI3K/Akt/mTOR pathway. Oncol Rep.

[42] Bu Y, Diehl JA (2016). PERK integrates oncogenic signaling and cell survival during cancer development. J Cell Physiol.

[43] Zhang TA, Zhang Q, Zhang J (2023). Identification of the role of endoplasmic reticulum stress genes in endometrial cancer and their association with tumor immunity. BMC Med Genomics.

[44] Lin S, Wei C, Wei Y, Fan J (2024). Construction and verification of an endoplasmic reticulum stress-related prognostic model for endometrial cancer based on WGCNA and machine learning algorithms. Front Oncol.

[45] Liu X, Song J, Liu H, Sun Z, Ren H, Luo J (2023). Endoplasmic reticulum stress could predict the prognosis of cervical cancer and regulate the occurrence of radiation mucositis. Dose Response.

[46] Zhang W, Shi Y, Oyang L (2024). Endoplasmic reticulum stress—a key guardian in cancer. Cell Death Discov.

[47] Guzel E, Arlier S, Guzeloglu-Kayisli O (2017). Endoplasmic reticulum stress and homeostasis in reproductive physiology and pathology. Int J Mol Sci.

[48] Silva VR, Neves SP, Santos LS, Dias RB, Bezerra DP (2020). Challenges and therapeutic opportunities of autophagy in cancer therapy. Cancers (Basel).

[49] Bergmann TJ, Fumagalli F, Loi M, Molinari M (2017). Role of SEC62 in ER maintenance: a link with ER stress tolerance in SEC62-overexpressing tumors?. Mol Cell Oncol.

[50] Taguchi Y, Horiuchi Y, Kano F, Murata M (2017). Novel prosurvival function of Yip1A in human cervical cancer cells: constitutive activation of the IRE1 and PERK pathways of the unfolded protein response. Cell Death Dis.

[51] Liu W, Zhang L, Shi J (2015). Clinical significance of pAkt and pErk1/2 expression in early-stage breast cancer patients treated with anthracycline-based adjuvant chemotherapy. Oncol Lett.

[52] Kim KW, Moretti L, Mitchell LR, Jung DK, Lu B (2010). Endoplasmic reticulum stress mediates radiation-induced autophagy by perk-eIF2α in caspase-3/7-deficient cells. Oncogene.

[53] Botrus G, Miller RM, Uson Junior PL, Kannan G, Han H, Von Hoff DD (2022). Increasing stress to induce apoptosis in pancreatic cancer via the unfolded protein response (UPR). Int J Mol Sci.

[54] Liang D, Khoonkari M, Avril T, Chevet E, Kruyt FA (2021). The unfolded protein response as regulator of cancer stemness and differentiation: mechanisms and implications for cancer therapy. Biochem Pharmacol.

[55] Adamczyk-Grochala J, Bloniarz D, Zielinska K, Lewinska A, Wnuk M (2023). DNMT2/TRDMT1 gene knockout compromises doxorubicin-induced unfolded protein response and sensitizes cancer cells to ER stress-induced apoptosis. Apoptosis.

[56] Zheng X, Andruska N, Lambrecht MJ (2016). Targeting multidrug-resistant ovarian cancer through estrogen receptor α dependent ATP depletion caused by hyperactivation of the unfolded protein response. Oncotarget.

[57] Bonsignore G, Martinotti S, Ranzato E (2023). Endoplasmic reticulum stress and cancer: could unfolded protein response be a druggable target for cancer therapy?. Int J Mol Sci.

[58] Wu Y, Wang G, Yang R (2025). Activation of PERK/eIF2α/ATF4 signaling inhibits ERα expression in breast cancer. Neoplasia.

[59] Lin J, Liu H, Fukumoto T (2021). Targeting the IRE1α/XBP1s pathway suppresses CARM1-expressing ovarian cancer. Nat Commun.

[60] Song M, Sandoval TA, Chae CS (2018). IRE1α-XBP1 controls T cell function in ovarian cancer by regulating mitochondrial activity. Nature.

[61] McMellen A, Yamamoto TM, Qamar L (2023). ATF6-mediated signaling contributes to PARP inhibitor resistance in ovarian cancer. Mol Cancer Res.

[62] Gray MJ, Mhawech-Fauceglia P, Yoo E, Yang W, Wu E, Lee AS, Lin YG (2013). AKT inhibition mitigates GRP78 (glucose-regulated protein) expression and contribution to chemoresistance in endometrial cancers. Int J Cancer.

[63] Xu S, Butkevich AN, Yamada R (2012). Discovery of an orally active small-molecule irreversible inhibitor of protein disulfide isomerase for ovarian cancer treatment. Proc Natl Acad Sci U S A.

